# A Study of Naturally Acquired Canine Babesiosis Caused by Single and Mixed *Babesia* Species in Zambia: Clinicopathological Findings and Case Management

**DOI:** 10.1155/2015/985015

**Published:** 2015-11-22

**Authors:** King Shimumbo Nalubamba, Ntombi Basimbi Mudenda, Mwaka Mwangala Namwila, Chilufya Susan Mulenga, Eugene Chisela Bwalya, Ethel M'kandawire, Ngonda Saasa, Careen Hankanga, Elizabeth Oparaocha, Martin Simuunza

**Affiliations:** ^1^Department of Clinical Studies, University of Zambia, School of Veterinary Medicine, P.O. Box 32379, 10101 Lusaka, Zambia; ^2^Vet-Serve Veterinary Practice, P.O. Box 38851, 10101 Lusaka, Zambia; ^3^Kaoma District Veterinary Offices, Department of Veterinary Services, Ministry of Agriculture and Livestock, Zambia; ^4^Nsama District Veterinary Offices, Department of Veterinary Services, Ministry of Agriculture and Livestock, Zambia; ^5^Graduate School of Veterinary Medicine, Laboratory of Surgery, Hokkaido University, Kita-18, Nishi-9, Kita-Ku, Sapporo 060-0818, Japan; ^6^Department of Disease Control, University of Zambia, School of Veterinary Medicine, P.O. Box 32379, 10101 Lusaka, Zambia; ^7^Showgrounds Veterinary Clinic, P.O. Box 30333, 10101 Lusaka, Zambia

## Abstract

A retrospective and prospective analysis of clinical records of dogs diagnosed with* Babesia* infections was carried out for the years 2000 to 2013 from practices in Lusaka, Zambia. Records of 363 dogs with confirmed* Babesia* infections were analysed using demographic factors including sex, breed, age, and clinical signs in relation to haematological findings and* Babesia* species. The clinical and laboratory findings observed are described as well as* Babesia* species identification. The study included 18 breeds and the highest proportion were mongrels (32.2%), males representing 64.5% of the population. The most common presenting problems were anorexia (65.3%) and lethargy/weakness (65.3%). The most common clinical signs were fever (87.3%), pallor (52.3%), lymphadenopathy (47.4%), and presence of ticks (44.9%). Anaemia (96.4%) and nucleated erythrocytes (42.2%) were the most common laboratory findings. A mixed infection of* Babesia rossi* and* Babesia gibsoni* was present in 59.7% of dogs, whilst 8% and 32.2% had* B. rossi* and* B. gibsoni* as a single infection, respectively. Case management mainly involved therapy with tetracyclines and imidocarb and was usually accompanied by clinical improvement. This study highlights, for the first time, the presence of* B. gibsoni* in natural dog populations in Zambia, where previously only* B. rossi* was reported.

## 1. Introduction

Babesiosis is a tick-borne disease, caused by protozoa of the genus* Babesia*, with a worldwide distribution [1, 2]. Canine babesiosis is an important disease of domestic and wild Canidae [3].* Babesia* are intraerythrocytic, vector-borne organisms with numerous canine* Babesia* species reported in the world, namely,* Babesia canis*,* Babesia rossi*,* Babesia vogeli*,* Babesia gibsoni*, and the microbabesiae* Babesia microti*,* Babesia vulpes *sp.* nov*, and* Babesia conradae* [4–6]. In Africa,* Babesia canis*,* B. rossi*,* B. vogeli*, and* B. gibsoni *are reported, with no report of the microbabesiae [4, 7].* Babesia rossi* is only reported in Africa [7]. There also exists one yet unnamed* Babesia* species that was reported in North America [4, 7].

The most common clinical signs associated with babesiosis are anorexia, fever, depression/lethargy, pale mucosae, splenomegaly, and weight loss [8–11]. Clinical presentation of canine babesiosis differs with geographic regions and* Babesia* species involved [4, 5, 12, 13]. Unusual clinical presentations of canine babesiosis that include central nervous system associated signs such as seizures have also been reported [14, 15]. In Africa, there is limited published data on canine babesiosis and outwith South Africa and Nigeria there are even fewer published data available [16]. There are numerous factors that affect the prevalence of canine babesiosis, and studies with* B. canis* have shown that pure-bred dogs, animals under one year of age, and dogs that had previously suffered from babesiosis or have not had prophylactic ectoparasite treatment are more prone [17]. Coinfection of* Babesia* with other canine vector-borne diseases occurs [16] and often leads to atypical clinical signs and presentations [18, 19].

Clinicopathological pictures in* Babesia* cases include regenerative anaemia, thrombocytopenia, and leukopenia (neutropenia and lymphopenia) initially after infection, soon followed by leukocytosis and neutrophilia with a left shift a few days after infection [20]. Chronic cases of canine babesiosis tend to present a more obscure range of clinical signs. Prognostic factors of* Babesia* infections in dogs include blood lactate concentration and PCV [21].* Babesia rossi *is hypothesised to have a polymorphic phosphoprotein named the* Babesia rossi* erythrocyte membrane antigen 1 (*BrEMA1*) gene that may be responsible for virulence [16]. This may explain some of the differences in clinical presentations in dogs infected with this species.

A number of therapeutic options are available for managing canine babesiosis. Imidocarb dipropionate, diminazene aceturate, clindamycin [7, 22], and doxycycline [23] are efficacious. Blood transfusion improves clinical outcome of therapy [24]. However, in cases of infection with small* Babesia* species, clinical and parasitological cure are often not attained and relapses are reported frequently [7]. Supportive treatment is usually given and includes fluid therapy, anti-inflammatory and antipyretics [20], gastroprotectants, oxygen supplementation, and blood transfusion [22]. In experimental* Babesia* infection, clinical recovery has been shown with supportive treatment only [20], highlighting the importance of this aspect of the case management.

Control involves the use of topically applied acaricides [25] to eliminate the tick vector and may also include novel methods like acaricide-impregnated collars [26]. The prophylactic use of imidocarb when animals move into endemic areas [10] can also be done. Vaccination against babesiosis has been developed [27] that induces partial protection against the disease by reducing severity of clinical signs, parasitaemia, or duration of clinical disease [7]. However, as with vaccination, most current prophylactic measures are considered insufficient for complete protection [5].

In Zambia, a pilot study was carried out to determine the epidemiology of canine* Babesia* infections in laboratory samples and natural populations; the study showed that canine* Babesia* is of the large-type and has a mean monthly prevalence of up to 28.6% and 2.4%, respectively, with two seasonal peaks in the rainy season and the cool dry season [28]. A recent molecular study in wildlife and domesticated dogs around wildlife reserves in Zambia found* Babesia* species in wild carnivores, but no* Babesia* in domestic dogs [29]. Despite the fact that knowledge of the clinicopathological manifestation of a disease such as babesiosis has epidemiological and medical importance [7], there is no published literature on the clinicopathological presentation of* Babesia* in dogs in Central Africa. The aim of this follow-up study was to document the clinical signs, haematological profiles, molecular speciation, and case management options in naturally occurring canine babesiosis in Zambia.

## 2. Materials and Methods

### 2.1. Inclusion Criteria

A mixed, retrospective, and prospective study of clinical cases of babesiosis was carried out. Cases were conveniently sampled and the inclusion criteria were dogs with complete, well-documented medical records, those that tested positive for* Babesia* on blood smear examination and had complete haematological profile. Clinical records from the Veterinary Clinic, School of Veterinary Medicine, University of Zambia, and two private veterinary practices in Lusaka for dogs that met the inclusion criteria were considered in this study. Dogs that had other diagnosed concurrent diseases or incompletely documented medical records were excluded from the study.

### 2.2. Case Recruitment

#### 2.2.1. Retrospective

Medical records of dogs diagnosed with* Babesia* from 2000 to 2009 were reviewed. Information on signalment, presenting problem, clinical signs, haematological findings, case management/treatment regimens, and case outcomes were documented. Data from the clinical records were entered into a data capture form.

#### 2.2.2. Prospective

The prospective study was carried out from March 2009 to December 2013. Data capture forms were filled in by veterinary clinicians for all cases that were clinically suspected to have the canine vector-borne disease commonly diagnosed as “tick fever.”

#### 2.2.3. Blood Collection and PCV

Blood collected by cephalic venipuncture was subjected to laboratory analysis as described previously [28] and dogs were classified as anaemic based on a modification of the classification criteria of Reyers et al. (1998) cited by Jacobson [14] combined with that of Tvedten and Weiss [30]. Dogs with PCV less than 15% were considered as severely anaemic, and those with PCV 15–30 as moderately anaemic, those with PCV >30–37 as mildly anaemic, and those with PCV >37% as nonanaemic.

Those that were positive on blood smear examination and had complete haematology performed were included in the study.

Ticks were also collected from each dog, placed in a plain 10 mL serum tube with 70% ethanol, and identified to genera level using the key by Soulsby [31].

### 2.3. DNA Extraction and Seminested PCR

Sixty-two* Babesia*-positive cases from the prospective study had their blood spotted on Whatman FTA cards for subsequent DNA extraction and* Babesia* species identification by seminested PCR using the method and primers used by Birkenheuer et al. [32].

DNA was extracted using the saponin lysis method and stored at −20°C until use. The seminested PCR was carried out as described by Birkenheuer et al. [32]. The primary PCR amplified an approximately 340-base-pair (bp) fragment from* B. gibsoni* (Asian genotype) and* B. rossi*. The secondary PCR using the specific internal primers for* B. gibsoni* (Asian genotype) (BgibAsia-F) and* B. rossi* (BCR-F) were paired with the outer reverse primer of the primary PCR to amplify 185 bp and 197 bp amplicons, respectively. PCR products were visualised in a transilluminator after electrophoresis in a 1.5% agarose gel containing 0.2 *μ*g Midori Green.

### 2.4. Data Handling and Analysis

Data were entered into a Microsoft Excel spreadsheet, verified for correctness, and imported into Minitab version 14 for statistical analysis and graphing. Statistically significant differences were defined as those with *P* ≤ 0.05. Continuous data were analysed using one-way analysis of variance (ANOVA). Tukey's multiple comparison* post hoc* test was used for pairwise comparisons. Pearson's correlation test was used to determine correlation. The Chi-square test was used to determine differences in proportions.

## 3. Results

From the 412 medical records of cases diagnosed as positive for* Babesia* on blood smear examination, 363 were included in the study having satisfied the inclusion criteria. The others were excluded from the study mostly because they either did not have a complete haematology profile and diagnosis was reached using a blood smear only or had incompletely filled in clinical records or data capture forms.

### 3.1. Dog Breeds, Age, and Gender Distribution

**Figure 1:**
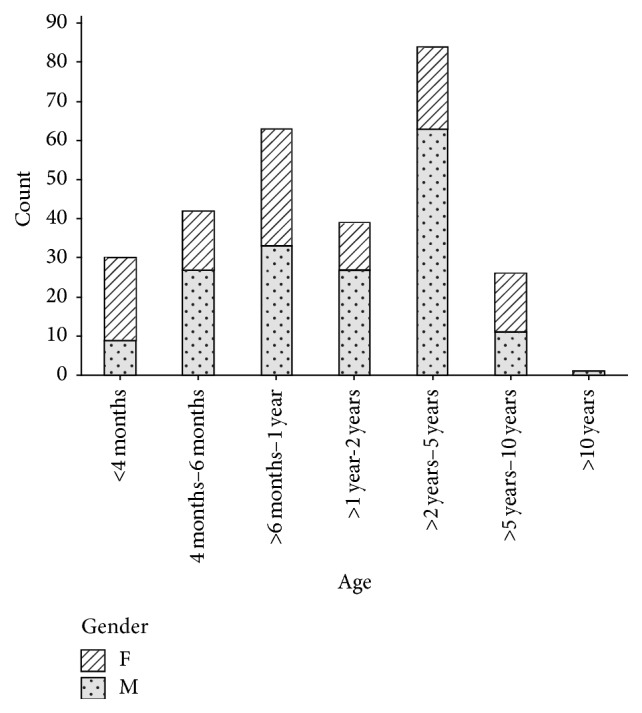
Age and gender distribution of the dogs included in the study.

Records for 363 patients were analysed and the most represented dog breeds were mongrels (32.2%) and Maltese poodles (16.3%) whilst the Shar Pei and Boston Terrier were the least represented (Table 1). Seventeen distinct exotic pure breeds and two other classes (pure-breed crosses and local breed/mongrels) were included. Thus, the exotic dogs and their crosses put together made up the largest proportion of dogs diagnosed with* Babesia* (Table 1). Male dogs made up the majority (64.5%) of the study subjects (Figure 1). Exact age was determined in 89% (323/363) of the dogs using the record inoculation card and ranged from two months to eight years (median age 18 months). As shown in Figure 1, dogs between two and five years made up the largest proportion (29.47%), followed by those in the six-month to one-year age bracket (22.11%). Dogs older than 5 years were the smallest group including one male dog that was more than 10 years old. The dogs whose exact ages could not be determined were only classified as adult dogs.

### 3.2. History and Physical Examination Findings

**Figure 2:**
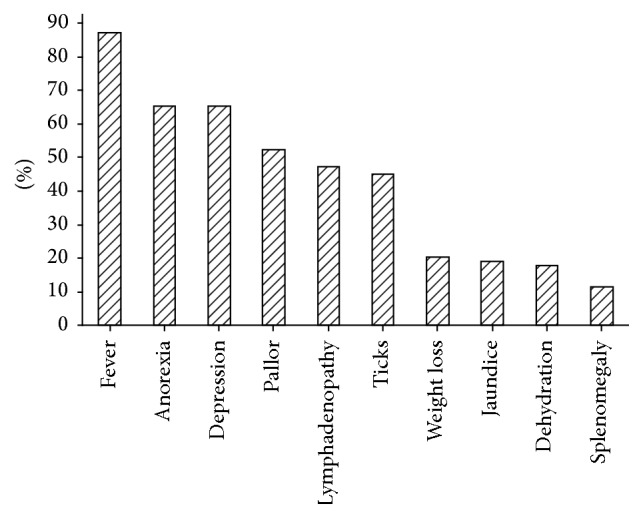
The ten (10) most commonly seen clinical signs and presenting problems associated with* Babesia* infection in dogs in Zambia (*n* = 363).

The different clinical signs and presenting problems are shown in Figure 2. The main problems reported by the owners were anorexia (65.3%), depression/lethargy (65.3%), and weight loss (20.4%). The most common clinical signs were fever (87.3%), pallor (52.3%), and lymphadenopathy (47.4%). Average rectal temperature was 39.6°C (±SD, range; ±0.995, 34.7–41.7). The study further demonstrated that 4.7% of the dogs had subnormal temperatures (temp. < 37°C). There was no significant difference in rectal temperature between local breed and exotic dogs (ANOVA: *F*_1, 319_ = 0.04; *P* = 0.84; exotic mean temperature ± SD = 39.58 ± 1.04, mongrel mean temperature ± SD = 39.62 ± 0.92). Forty-five percent of the dogs were infested with ticks mainly of the genus* Rhipicephalus*. Other clinical signs/problems observed included vomiting, splenomegaly, dehydration, jaundice, and ascites. Two cases presented with severe epistaxis. Only a single case showed seizures with polyuria/polydipsia. There was a weak positive significant correlation between PCV and rectal body temperature (*r* = 0.231, *P* = 0.017).

### 3.3. Laboratory Findings

#### 3.3.1. Parasite Identification

Microscopic examination confirmed that all the dogs were infected with* Babesia *species. PCR reaction was able to differentiate* B. gibsoni* (Asian genotype) as a single infection in 32.2% (20/62) and* B. rossi *as a single infection in 8% (5/62) of the samples. Most (37/62; 59.7%) of the other samples had a mixed infection.

#### 3.3.2. Haematological Findings

**Figure 3:**
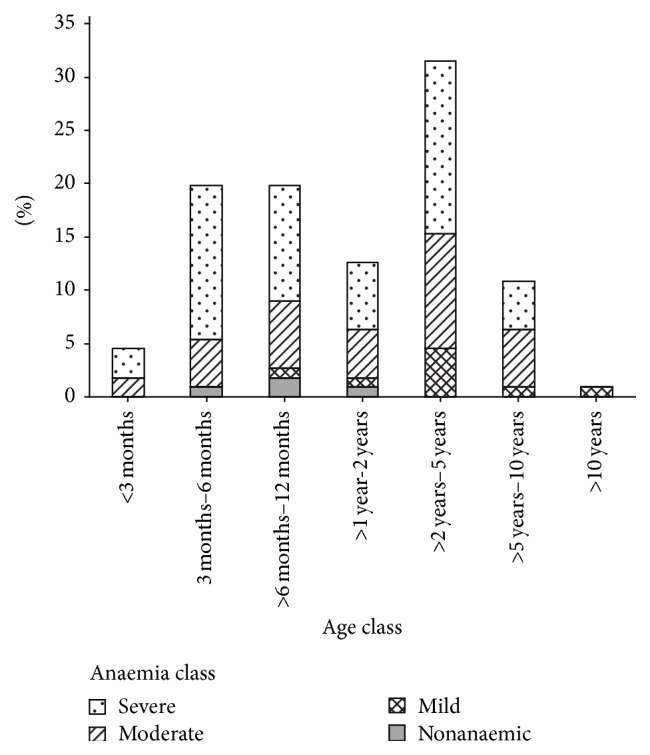
Severity of anaemia in* Babesia* infected dogs by age class. Anaemia was classified as severely anaemic (PCV less than 15%); moderately anaemic (PCV 15–30); mildly anaemic, (PCV > 30–37); and nonanaemic (PCV > 37%).

**Figure 4:**
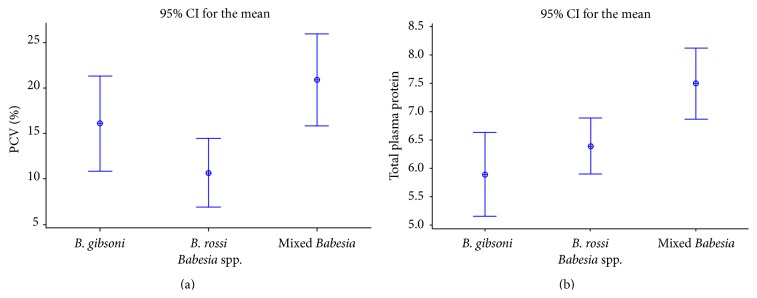
Interval plots of packed cell volume values (a) and total plasma proteins (b) in dogs with single and mixed* Babesia* species infections.

Table 2 outlines the haematological findings in blood samples. The most consistent haematological abnormalities were anaemia (96.4%), nucleated erythrocytes (42.2%), and hypochromasia (34.7%). The anaemia was hyporegenerative, normocytic, and normochromic in 65.3% of the cases (Table 3). Overall mean PCV was 18.4% (±SD, range; ±9.2, 5.0–49.0) and overall mean Hb was 6.4 g/L (±SD, range; ±3.04, 1–14.1). Severe anaemia was seen in 55.1% of dogs, whilst 33% showed moderate anaemia, 8.0% showed mild anaemia, and 3.6% were nonanaemic (Figure 3). There were also no differences in proportions of dogs showing severe anaemia by age class (Figure 3). However, only the age groups between three months and two years had some* Babesia*-positive dogs with no signs of anaemia, whilst in the other age groups they were all anaemic (Figure 3). Dogs with single infections with* B. rossi* had lower PCVs and Hb levels, followed by those with a single infection with* B. gibsoni. *Dogs with concurrent infection with both* B. gibsoni* and* B. rossi* had higher PCVs and Hbs than those with single infections of either, but the differences were not significant (ANOVA: *F*_2, 60_ = 2.03; *P* = 0.15;* B. rossi* mean PCV ± SD 10.67 ± 1.53;* B. gibsoni* mean PCV ± SD 16.11 ± 6.81; mixed infection PCV ± SD 20.15 ± 9.94) (Figure 4). Thrombocytopenia was observed in 17.1% of the cases. There were no statistical differences in PCV between mongrels and exotic breeds and their crosses (ANOVA: *F*_1, 362_ = 0.02; *P* = 0.876; exotic mean PCV ± SD 18.28 ± 8.94, mongrel mean PCV ± SD 18.5 ± 9.62).

### 3.4. Case Management and Case Outcomes

Cases were often treated with the injectable and oral drugs outlined in Table 4. Imidocarb dipropionate together with doxycycline was the most commonly used specific antiprotozoal injectable treatment (51.0%) followed by doxycycline only (24.0%) and imidocarb dipropionate only (5.5%), and only 0.8% (3/363) of cases were managed with diminazene as an antibabesial drug (Table 4). Approximately sixty percent (219/363) were treated with a 10–21-day course of oral doxycycline at 10 mg/kg body weight twice a day.

Adjunct immunosuppressive treatments were given to some dogs (a total 37.4% given dexamethasone injection, 5.1% (20/363) via injectable dexamethasone and oral prednisolone, and 1.4% (5/363) given oral prednisolone only). Other treatments included the use of gastroprotectants (0.8% omeprazole, 2.2% sucralfate). Blood transfusions were performed in 1.9% (7/363) of the cases with six of the seven cases being the hospitalised ones. Approximately 27% (97/363) of the dogs were given topical ectoparasitic treatment that included dipping (11.6%), spot-on treatments (8.5%), and sprays (6.6%).

Of the dogs diagnosed with* Babesia*, 41.9% (152/363) were hospitalised, whilst the rest were treated on an outpatient basis. Hospitalisation was on average 1.74 ± 2.15 days and ranged from mostly overnight observation to a maximum of 21 days. There was no significant difference in the proportion of anaemia and PCV in dogs that were hospitalised and those that were not (ANOVA: *F*_1, 362_ = 5.25;  *P* = 0.030; hospitalised mean PCV ± SD = 15.36 ± 6.18, outpatient mean PCV ± SD = 21.36 ± 7.60). Hospitalised dogs were more likely to be the ones given a blood transfusion than outpatient cases (*χ* ^2^ = 5.636, *df* = 1, and *P* = 0.018) noting the* caveat* that the outpatient group only had one blood transfusion case, which is less than the minimum five normally recommended for the Chi-square test.

## 4. Discussion

The present study describes the clinical signs and case management of naturally occurring babesiosis in Zambia. The study represents the first systematic investigation of the clinicopathological presentation of canine babesiosis in this region as well as reporting for the first time the species of* Babesia* found in domestic dogs in Zambia in correlation to clinicopathological data. The study presents new information about the clinicopathological presentation of canine babesiosis and the* Babesia* species involved, namely,* B. gibsoni* and* B. rossi*. Although the lack of positive cases in domestic dogs in a recent molecular survey in Zambia [29] may be ascribed to regional differences between the central part of Zambia and the eastern and western regions where the recent study [29] was conducted. The aforementioned study also had a sample size of only eight domestic dogs sampled, which may have an effect on the results, taking into consideration the low prevalence of* Babesia* in natural domestic dog populations in Zambia of 2.4% [28]. A similar low canine* Babesia* prevalence of 3.8% was also reported in Cape Verde [34]. A more widespread investigation using a larger number of dogs from all around the country would be more desirous in order to comprehensively document clinicopathological presentation of this disease and determine if there are regional differences in* Babesia* prevalence [17] that may be ascribed to differences in* Babesia* parasites species or vector distribution as has been shown by other researchers [35].

The most common clinical problems for dogs with* Babesia* in this study were fever, depression, pallor, lymphadenopathy, and the presence of ticks. Fever was a consistent finding in dogs with* Babesia* infections in this study with 87.3% having fever and 47.4% exhibiting lymphadenopathy. A high proportion of dogs in this study presented with a fever of more than 40°C which is similar to the fever of 40.4°C as described by Casapulla et al. [36] in a* B. gibsoni* infected dog. Unlike findings by Adaszek et al. [37], Salem and Farag [38], and Davitkov et al. [11], who found changes in urine colour and haemoglobinuria, respectively, as being a prominent clinical finding, and the single unusual case by Demeter et al. [39] that presented with haemoglobinuria, this study did not have a case presenting with haemoglobinuria. This, however, could be due to the fact that this clinical sign is not commonly seen due to the fact that urine is not passed, neither is it collected at each and every clinical examination. Further, most owners may not be very observant or may not see the dog passing discoloured urine and therefore the finding may be underreported. Microscopic haemoglobinuria may have also been present in the absence of grossly visible macroscopic haemoglobinuria.

Most dogs were infested with ticks of the genus* Rhipicephalus* and there were no statistical differences in infestations between male and female dogs. The higher prevalence of genus* Rhipicephalus* is similar to findings by Abd Rani et al. [3] in India that also has a tropical climate like Zambia. Notwithstanding this fact, a number of animals presented with* Babesia* without any ectoparasites and a history of consistent tick control. This could be attributed to modified history proffered by owners due to a possible guilt-feeling combined with a possible recent deticking, prior to presentation to a veterinary practice, by the owners when animals appeared to be falling ill. However, a similar finding of approximately only half of the animals being diagnosed with* Babesia* infection being infested with ticks was reported by Cardoso et al. [40] in Portugal.

In a similar vein, in the prospective study, a number of animals that were clinically suspected to have babesiosis by clinicians were microscopically negative for* Babesia*. This could be due to a number of reasons: the inherent low sensitivity of the blood smear microscopy compared to molecular biological tests like PCR; parasitaemia is also transient in infected animals, particularly early in infection, and low and not detectable in chronic cases [5]. However, since the study was designed to describe the clinicopathological findings in* Babesia*-positive dogs, the only drawback was the reduced sample size due to possible false-negatives arising due to the aforementioned reasons. The use of PCR would have likely led to a larger sample size.

Anaemia, nucleated erythrocytes, and thrombocytopenia have been demonstrated as consistent laboratory findings in canine babesiosis by numerous researchers [37, 38, 41] and this study also revealed that anaemia is consistent in dogs with* Babesia* but thrombocytopenia was only seen in less than one-fifth of the dogs.

Overall the clinical-pathological findings from this study are comparable with findings by many researchers such as Ruiz de Gopegui et al. [13] other than the haemoglobinuria which was not observed as a prominent clinical sign in this study.

This study would seem to indicate that single infections with* B. rossi* or* B. gibsoni* give more severe haematological results than mixed infection. The authors could ascribe no particular reason to this paradoxical finding. Other researchers have also described* Babesia* species coinfections but also state that it is difficult to ascribe clinical signs to a single specific agent [41]. Similar to Salem and Farag [38] more male dogs than female were captured in this study. Adaszek et al. [17] found that male dogs are more prone to infection with* Babesia* infection.

Differences between local breed dogs and exotics have been demonstrated where the exotics were more prone [17]. This study, however, did not show any differences in clinicopathological findings between the two groups. Similar to Adaszek et al. [17], this study also found that dogs less than one year were more prone to infection with* Babesia*.

Our study shows that more animals have coinfection with both* B. gibsoni* and* B. rossi* and only a few have a single infection with either species. This is in contrast with findings of coinfection with* B. canis *and* B. vogeli* by Manzillo et al. [41] in Italy and Kamani et al. [42] with* B. canis* and* B. rossi* in Nigeria. The proportion of coinfections in this current study is, however, higher than reported by Adamu et al. in Nigeria [16]. There was a higher proportion of dogs with* B. gibsoni* as a single infection than those with* B. rossi* as a single infection. Due to the fact that most animals had coinfections it was difficult to ascribe clinical signs and haematological abnormalities to a single* Babesia* species [41]. Other researchers in Africa [38, 42, 43] and Cape Verde [34] did not find* B. gibsoni* and in South Africa it is only reported in an imported dog [44].

Babesiosis may be classified as complicated or uncomplicated with complicated babesiosis manifesting as an acute, serious life-threatening disease [4]. The findings from this study, therefore, demonstrate that the canine babesiosis in Zambia is less virulent/acute and possibly classified as uncomplicated than that described in South Africa [14]. However, the increased trade in dogs originating from South Africa increases the possible risk of introduction of more virulent strains of* Babesia* into Zambia since travelling dogs have been shown to introduce* Babesia* pathogens in Germany [45] and South Africa [44].* B. vogeli* that has been reported in South Africa [44], Egypt [38], and Cape Verde [13] has not been reported in Zambia. The limiting factor in this study was the fact that the primers used in this study were not specific for* B. vogeli*. Further, the South African study [46] did not report mixed infection between* B. rossi* and* B. vogeli*, but our study demonstrated a high proportion of dogs with a mixed infection of* B. rossi* and* B. gibsoni*.

Patterns of drug use also demonstrate changing trends with diminazene that was being used during the earlier part of the decade seldom being used with most practices exclusively using imidocarb dipropionate as the antibabesial drug of choice. No other anti-*Babesia* drug such as clindamycin and quinine, and atovaquone-azithromycin, reported in literature [22] was used in the cases in the study.

In conclusion, this study highlights the species of canine* Babesia* present in Zambia as well as the clinicopathological findings in canine babesiosis. It also highlights differences between canine babesiosis disease presentation in Zambia and other countries in the subregion.

## Figures and Tables

**Table 1:** Distribution of breeds of dogs included in this study.

Breed	Count
Boerboel	16
Boston Terrier	1
Boxer	4
Cocker Spaniel	2
Crossbreed^*∗∗*^	43
Doberman	8
Great Dane	2
GSD	51
Jack Russell	10
Labrador	6
Maltese poodle	59
Mastiff	1
Mongrel	117
Pomeranian	16
Ridgeback	4
Rottweiler	17
Shar Pei	1
Staffordshire Terrier	2
Yorkshire Terrier	3

Total	363

^*∗∗*^Crossbreed dogs are dogs classified as such because they have one known distinct exotic breed but are crossed with another breed dog.

GSD: German shepherd dog.

**Table 2:** Overall haematological findings in blood samples from *Babesia*-positive dogs (*n* = 363) and by species in Zambia.

Parameter	All *Babesia*-positive (mean ± SD)	*B. rossi* (mean ± SD)	*B. gibsoni* (mean ± SD)	Mixed (mean ± SD)	Reference values [33]
WBCs × 10^3^/*μ*L	9.67 ± 7.46	17.17 ± 8.46	8.68 ± 3.78	9.04 ± 6.32	5.5–16.9
RBCs × 10^6^/*μ*L	2.98 ± 1.50	1.74 ± 0.44	2.92 ± 1.06	3.17 ± 1.40	5.5–8.5
PCV (%)	18.37 ± 9.22	10.67 ± 1.53	16.11 ± 6.81	20.15 ± 9.94	37–55
P/protein (g/dL)	7.15 ± 1.28	6.40 ± 0.20	5.90 ± 0.88	7.48 ± 1.20	5.4–7.7
Haemo'gb (g/dL)	6.40 ± 3.05	4.07 ± 0.23	5.26 ± 2.69	6.86 ± 3.33	12.0–18.0
MCV (fL)	64.19 ± 13.34	62.73 ± 8.09	62.16 ± 4.55	63.51 ± 4.45	60–72
MCHC (%)	35.94 ± 6.14	38.70 ± 6.85	33.44 ± 1.20	33.79 ± 1.28	31–37
MCH (pg)	23.35 ± 5.30	24.67 ± 7.74	20.60 ± 1.99	21.45 ± 1.77	19.5–24.5

*WBC differential count*					
Band neutrophils %	3.29 ± 4.80	6.00 ± 1.73	1.80 ± 0.84	3.44 ± 2.31	0–3
Seg. neutrophils %	55.98 ± 13.24	58.0 ± 23.5	50.60 ± 20.34	55.12 ± 14.69	60–80
Lymphocytes %	29.57 ± 11.45	26.0 ± 24.3	38.20 ± 20.96	31.29 ± 12.70	10–34
Eosinophils %	4.26 ± 2.38	4.00 ± 1.00	4.20 ± 3.03	4.765 ± 1.52	2–10
Monocyte %	6.73 ± 4.48	4.87 ± 3.74	6.65 ± 4.73	5.93 ± 4.18	1–11
*n*	363	5	20	37	

**Table 3:** Other haematological findings from blood samples from *Babesia*-positive dogs (*n* = 363) in Zambia.

Laboratory comment	*n*	Positive %
NRBCs	154	42.42
Anisocytosis	79	21.76
Crenated RBCs	43	11.85
Hypochromasia	126	34.71
Increased platelets	36	9.92
Thrombocytopenia	62	17.08
Lymphocytosis	79	21.76
Monocytosis	60	16.53
Leukopenia	19	5.23
Neutrophilia	9	2.48

NRBC: nucleated red blood cell; RBC: red blood cell.

**Table 4:** Most commonly used therapeutic agents to manage cases of canine babesiosis in Zambia.

Drug	*n*	%
*Injectable*		
Antipyretic (NSAIDs)	17	4.68
B complex	152	41.87
Blood transfusion	7	1.93
Dexamethasone	136	37.47
Diminazene	3	0.83
Doxycycline only	87	23.97
Doxycycline & imidocarb	185	50.96
Imidocarb only	20	5.51
Intravenous (IV) fluids	15	4.13
Ivermectin	19	5.23
Oxytetracycline (OTC)	5	1.38
OTC plus imidocarb	11	3.03
*Oral drugs*		
B complex	173	47.66
Doxycycline	219	60.33
Prednisolone	25	6.89
Sucralfate	8	2.20
Omeprazole	3	0.83
*Topical*		
Antiparasitic spray	24	6.61
Antiparasitic spot-on	31	8.54
Antiparasitic wash/dip	42	11.57

NSAIDs: nonsteroidal anti-inflammatory drugs.
